# Post-Event Processing After Embarrassing Situations: Comparing Experience Sampling Data of Depressed and Socially Anxious Individuals

**DOI:** 10.32872/cpe.v2i4.2867

**Published:** 2020-12-23

**Authors:** Jasmin Čolić, Anna Latysheva, Tyler R. Bassett, Christian Imboden, Klaus Bader, Martin Hatzinger, Thorsten Mikoteit, Andrea Hans Meyer, Roselind Lieb, Andrew T. Gloster, Jürgen Hoyer

**Affiliations:** a Institute of Clinical Psychology and Psychotherapy, Technische Universität Dresden, Dresden, Germany; b Private Clinic Wyss, Muenchenbuchsee, Switzerland; c Centre for Psychosomatics and Psychotherapy, Psychiatric Hospital, University of Basel, Basel, Switzerland; d Psychiatric Services Solothurn, Solothurn, Switzerland; e Centre for Affective, Stress and Sleep Disorders, Psychiatric Hospital, University of Basel, Basel, Switzerland; f Division of Clinical Psychology and Epidemiology, Department of Psychology, University of Basel, Basel, Switzerland; g Division of Clinical Psychology and Intervention Science, Department of Psychology, University of Basel, Basel, Switzerland; Philipps-University of Marburg, Marburg, Germany

**Keywords:** post-event processing, social anxiety, depression, transdiagnostic processes, embarrassment, experience sampling

## Abstract

**Background:**

Post-event processing (PEP) after social interactions (SIs) contributes to the persistence of social phobia (SP). This study investigated whether PEP as a transdiagnostic process also occurs in major depressive disorder (MDD) and controls. We also tested to what extent PEP was explained by trait levels of social anxiety (SA) or depression.

**Method:**

For seven days, a total of n = 165 patients (n = 47 SP, n = 118 MDD) and n = 119 controls completed five surveys per day on their smartphones. Event-based experience sampling was used. PEP was assessed following subjective embarrassment in SIs with two reliable items from the Post-Event Processing Questionnaire. Data were analysed via multilevel regression analyses.

**Results:**

Individuals with SP or MDD experienced more embarrassing SIs than controls and, accordingly, more PEP. The relative frequency of PEP after embarrassing SIs was equally high in all groups (86-96%). The groups did not differ regarding the amount of time PEP was experienced. After controlling trait depression, embarrassment occurred more frequently only in SP compared to controls. When controlling trait SA, between-group differences in indications of embarrassment, and consequently in PEP, dissipated.

**Conclusions:**

PEP could be interpreted as a common coping strategy among all individuals, while more frequent embarrassment might be specific for clinical groups. Embarrassment was primarily driven by SA. The alleviation of SA could lead to the reduction of embarrassment and, further, of PEP. On this basis, a model describing PEP in MDD is proposed, while current models of PEP in SP are complemented.

## Abstract (highlights)

- Individuals with social phobia or major depression experienced more embarrassing social interactions than healthy controls and, accordingly, more post-event processing.
- The frequency of post-event rumination within embarrassing interactions was high in all groups (86-96%).
- After controlling trait levels of social anxiety, between-group differences in the number of embarrassing situations, and consequently in post-event processing, dissipated.
- When controlling trait levels of depression, post-event rumination was higher in social phobia compared to healthy controls and major depression.

## Background

Social phobia (SP, or social anxiety disorder) is characterised by fear of acting in a way that could cause embarrassment or rejection from others in one or more social situations (APA, 2013). SP is highly persistent and usually has a chronic and stable course (Beesdo‐Baum et al., 2012; Fehm, Beesdo, et al., 2008). One of the key processes that contributes to its persistence is post-event processing (PEP; Brozovich & Heimberg, 2008; Clark & Wells, 1995; Hofmann, 2007; Rapee & Heimberg, 1997).

PEP refers to ruminative thinking that centres on one’s self-perception and anxious feelings following a social event (Abbott & Rapee, 2004; Clark & Wells, 1995). It is highly associated with in-situation anxiety and with avoidance of future social situations (Dannahy & Stopa, 2007; Hofmann, 2007; Mellings & Alden, 2000; Rachman et al., 2000). During PEP, the affected individual mentally reviews a previous event in detail, while pondering over thoughts indicative of the belief that he or she was evaluated negatively (Abbott & Rapee, 2004). This leads to the event being recalled as more negative than it actually was (Hofmann, 2007). Accordingly, PEP serves as a chain link in a vicious cycle in which recollections of past “failures” lead to anticipatory anxiety and to predictions of negative evaluation in subsequent social events (Mellings & Alden, 2000), thus increasing the probability to avoid such events completely (Rachman et al., 2000). Therefore, interventions designed to minimise PEP were included in prominent treatment protocols for SP (e.g. Rapee & Heimberg, 1997).

Because individuals with SP predominantly fear scrutiny by others, social situations in which said persons felt embarrassed or humiliated could bear particular risk for heightened social anxiety (SA) and PEP. In social interactions (SI), embarrassment usually results from *unwanted exposure* of a topic or motive that a person would rather keep hidden or concealed from others (Crozier, 2001). To avoid such exposure, individuals with SP maintain high self-focused attention, while scanning their environment for impending negative evaluation. They usually detect such signs rapidly, deeming their behaviour as embarrassing (Bögels & Mansell, 2004; Rapee & Heimberg, 1997). Both negative evaluation by others (Makkar & Grisham, 2011) and negative self-evaluation (Chen et al., 2013; Perini et al., 2006) have been shown to significantly predict PEP. Thus, embarrassment, as a catalyst for perceived negative evaluation, might significantly contribute to PEP.

Patterns of ruminative thinking such as PEP, are however symptomatic for many mental disorders (McEvoy et al., 2010). This is due to shared cognitive and behavioural processes underlying a wide range of clinical conditions (Ehring & Watkins, 2008; Harvey et al., 2004). Hence, it remains unclear whether PEP is specific to only SP.

Another disorder to which ruminative thinking has a robust and consistent relationship is major depressive disorder (MDD; Mor & Winquist, 2002; Nolen-Hoeksema et al., 2008). In MDD, rumination is defined as a response style that consists of repetitive and negative thinking about causes and implications of depressive symptoms (Nolen-Hoeksema, 1991; Nolen-Hoeksema et al., 2008). Rumination is associated with dysphoric mood in MDD (Nolen-Hoeksema, 2000; Nolen-Hoeksema & Morrow, 1993), and is predictive of the onset and duration of future depressive episodes (Nolen-Hoeksema, 2000; Nolen-Hoeksema et al., 1993). Rumination exacerbates and maintains depression by interfering with effective problem solving and with instrumental behaviour (Lyubomirsky & Nolen-Hoeksema, 1993, 1995; Nolen-Hoeksema et al., 2008; Pyszczynski & Greenberg, 1987). Unlike PEP in SP though, rumination in MDD is not bound to specific social events, but rather presents a more general, trans-situational style of thinking (McEvoy et al., 2010). Also, it revolves around depressive symptoms and themes of loss (Nolen-Hoeksema et al., 2008), while PEP in SP is related to social anxiety and thoughts of negative evaluation (Kocovski & Rector, 2007). However, as patients with MDD exhibit pronounced interpersonal problems as well (e.g. Garrison et al., 2012; Pemberton & Fuller Tyszkiewicz, 2016), this opens the possibility that they, just like socially anxious individuals, would also engage in PEP after social events.

In interpersonal encounters, depressed individuals were shown to be inhibited, reassurance seeking, and less socially skillful (Allen & Badcock, 2003; L. H. Brown et al., 2011; Hames et al., 2013; Joiner et al., 1999). This leads others to behave towards them in a more detached manner during the interaction or to avoid them completely (Gotlib et al., 2004; Segrin, 2000). Rejection by others can lead to feelings of loneliness and heightened dysphoric mood, which ultimately can lead to rumination (Hames et al., 2013; Heinrich & Gullone, 2006).

Individuals with MDD also have the propensity to process interpersonal reactions in a negative manner, even if they were not inherently harmful (Bistricky et al., 2016; Joiner et al., 1999). As embarrassing SIs are often accompanied by a certain reaction from others, like an evaluative gaze (Robbins & Parlavecchio, 2006), they could as well be potentially detrimental for individuals with MDD. Behaviours like that could be highly ambiguous and be appraised as negative evaluation (Gotlib et al., 2004; Joiner et al., 1999; Trew & Alden, 2009). Perceived negative evaluation can trigger depressive feelings and successive rumination in individuals with MDD, especially when it is linked to people close to the individual (like family members or partners; Anderson et al., 1999; Garrison et al., 2012). Hence, it can be assumed that feelings of embarrassment in SIs, once they are triggered, can produce ruminative thinking in depressed individuals.

One major methodological problem of the studies cited is recall bias, which refers to systematic errors during the retrieval of autobiographical episodes (Shiffman et al., 2008). Recall bias is especially accentuated in individuals prone to ruminative thinking (Williams et al., 2007), like individuals diagnosed with SP or MDD. These individuals tend to resort to *overgeneral memory* (Conway & Pleydell-Pearce, 2000) and tend to have difficulties recalling specific episodes. As a result, research methodologies that limit recall bias are needed. Experience sampling method (ESM) as a data collection strategy that is anchored in daily life has proven to bypass these limitations (Fahrenberg et al., 2007). While PEP in SP has successfully been investigated in everyday life (Badra et al., 2017; Helbig-Lang et al., 2016), no study to date has used ESM to explore whether PEP is a transdiagnostic phenomenon occurring in MDD as well. The findings could advance the understanding of the genesis, the predecessors and the clinical specificity of PEP, and shine light on its natural occurrence in everyday life. It would provide insights into social behaviour of individuals with MDD and the transdiagnostic character of PEP as well, which could contribute to the development and enhancement of appropriate treatment strategies.

On this basis, we explored the frequency and duration of PEP after embarrassing SIs in patients with SP and MDD, as well as controls without SP or MDD. We derived two main hypotheses. The first hypothesis (H1) concerned between-group differences in frequency and duration of PEP. Because PEP is primarily linked to SA and social situations (Fehm et al., 2007), and because of the higher importance of embarrassment in SP, we hypothesized that the frequency and duration of PEP would be significantly higher in SP compared to MDD. Due to symptoms of SA or depression being elevated in both MDD and SP, however, we also hypothesized that the frequency and duration of PEP would be significantly higher in both clinical groups (SP and MDD) compared to controls. The second hypothesis (H2) concerned the contribution of trait SA and trait depression to indications of embarrassment and to PEP. Due to the previously exemplified relation of dysphoric feelings to interpersonal rejection (e.g. Gotlib et al., 2004) we expected that PEP in MDD would be primarily driven by trait levels of depression, while PEP in SP would be facilitated by trait SA. To test this hypothesis, we analysed between-group differences while partialling out trait SA or trait depression. We expected that after controlling trait depression, PEP would remain elevated in SP compared to controls. On the other hand, when controlling trait SA, we expected that PEP would remain significantly higher in MDD compared to controls. Lastly, in our third hypothesis (H3) we explored if there are differences in embarrassment and PEP between the comorbid SP/MDD group and the SP group without MDD as comorbidity, and the MDD group without SP as a comorbid diagnosis. Because of elevated levels of *both* depression and SA, we predicted that PEP would be significantly higher in the comorbid group compared to the non-comorbid groups. We tested all our hypotheses in an ESM framework to minimise recall bias and to enhance ecological validity.

## Method

### Study Design

The study was part of a larger project about daily symptom fluctuations in MDD and SP (Gloster et al., 2017). Data collection was conducted at two research centers, one in Switzerland and one in Germany. Financing was provided by the Swiss National Science Foundation. The study protocol was approved by the ethics committee of the University of Basel (Approval # EKBB 236/12).

### Participants

#### Recruitment and Selection Criteria

Participant recruitment and data collection occurred between May 2014 and August 2016 (Gloster et al., 2017). Patients with SP and MDD were recruited through the outpatient clinics of the research centres, through local practitioners and through internet advertisements. If the recruited individuals were 18-65 years old, met diagnostic criteria for SP or MDD according to the Diagnostic and statistical manual of mental disorders (4^th^ ed., text rev., DSM-IV-TR; APA, 2000), and did not meet any of the exclusion criteria, they were invited to participate in the study. The diagnostic assessments were conducted with the Structured Clinical Interview for DSM-IV Axis I Disorders (SCID-I; First et al., 1997). The exclusion criteria were: current suicidal tendencies, current substance abuse and physical disabilities that prohibited proper use of a smartphone (e.g. an inability to see text on the screen or hear the smartphone’s signal; Gloster et al., 2017). The inability to understand German was exclusionary. The controls were recruited through internet advertisements. If, according to the SCID-I, they did not meet criteria for SP or MDD and were 18-65 years old, while not meeting any exclusion criteria, they were eligible for inclusion.

#### Sample Size Calculation

The outpatient clinics, from which the patients were recruited, see an estimated 110 SP and 520 MDD patients per year. Thus, the sample size calculation of the overall project (Gloster et al., 2017), in which the present study was embedded, was grounded on the assumption that the maximum number of patients with SP that could feasibly be recruited within the study time period would be *n* = 48. Assuming a dropout rate of 5%, this led to an expected number of 45 SP patients to complete the study. This number was used for the power analysis which assumed alpha = .05, power = .8, and a two-sided test for group comparisons on the between-subjects level. Based on a medium effect size (*d* = 0.5), and 45 subjects in the SP group, the sample size necessary to achieve .8 power is 111 subjects in each of the other groups (MDD & controls). Assuming a 5% dropout rate, 117 subjects would need to be recruited in each of these two groups. Given that we conducted multilevel analyses on the within-subjects level, which usually requires a smaller number of subjects to reach a certain degree of statistical power than the between-subjects level (Bellemare et al., 2016; Charness et al., 2012), we considered this sample size sufficient for the test of our hypothesis.

#### Final Sample

A total of *N* = 290 participants were initially included, but *n* = 6 of them did not complete at least 50% of the ESM time points. As an a priori decision (Gloster et al., 2017), these participants were removed from the dataset. The final sample size consisted of *N* = 284 (*n* = 119 controls; *n* = 118 with MDD; *n* = 47 with SP). In the SP group, *n* = 15 (31.9%) had co-morbid MDD, while *n* = 29 (24.6%) patients with MDD had co-morbid SP. In controls, *n* = 9 subjects fulfilled criteria for a clinical diagnosis. The sociodemographic and clinical characteristics of the sample, as well as prevalent diagnoses among controls are presented in Table 1.

**Table 1:** Sociodemographic and Clinical Characteristics of the Sample (N = 284)

Characteristics	Controls (*n* = 119)	MDD (*n* = 118)	SP (*n* = 47)
**Age (*M*, *SD*)**	32.2 (12)	32.7 (12)	28.3 (7.8)
**Female (%)**	67.2	66.1	66.0
Education (Years) (%)			
8-10	12.0	21.1	9.3
11-13	53.0	51.4	67.4
14+	35.0	27.5	23.3
Living arrangement (%)			
Alone	30.3	22.9	21.3
Family/partner	49.6	60.2	55.3
Other	20.2	16.9	23.4
**Employed (%)**	57.1	52.5	38.3
Number of diagnoses (%)			
0	90.8^a^	0.0	0.0
1	6.7	45.8	44.7
2	1.7	29.7	27.7
3+	0.8	24.6	27.7
**In therapy (%)**	14.3	58.5	46.8

*Note.* Controls = Control group; MDD = Major depressive disorder; SP = Social phobia.

^a^Following diagnoses were prevalent in controls: Specific phobia (*n* = 3), Panic disorder (*n* = 2), Anxiety disorder, unspecified (*n* = 1), Obsessive-compulsive disorder (*n* = 2), Agoraphobia with panic disorder (*n* = 1).

### Measures

#### Post-Event Processing

PEP was measured with two items from the Post-Event Processing Questionnaire (PEP-Q; Rachman et al., 2000; German Version: Fehm, Hoyer, et al., 2008): 1. *“Do you still think about the embarrassing moment from the interaction?”*; and 2. *“Do you have difficulties to forget the embarrassing moment?”*. The items were rated on a scale from 0 = *not at all* to 100 = *100% of the time since the interaction* (50 = *50% of the time*). The anchors of the scale were changed to percentages because the “percentage of time” approach is preferable to asking for durations, when symptoms lack a clear beginning or end (Schimmack et al., 2000). These items were chosen because of their high factor loadings on the first factor (Fehm, Hoyer, et al., 2008). The German version of the PEP-Q had an internal consistency of α =.72 in the original translation of the PEP-Q and α = .90 in the extended version (see Fehm, Hoyer, et al., 2008).

#### SIs

**Figure 1:**
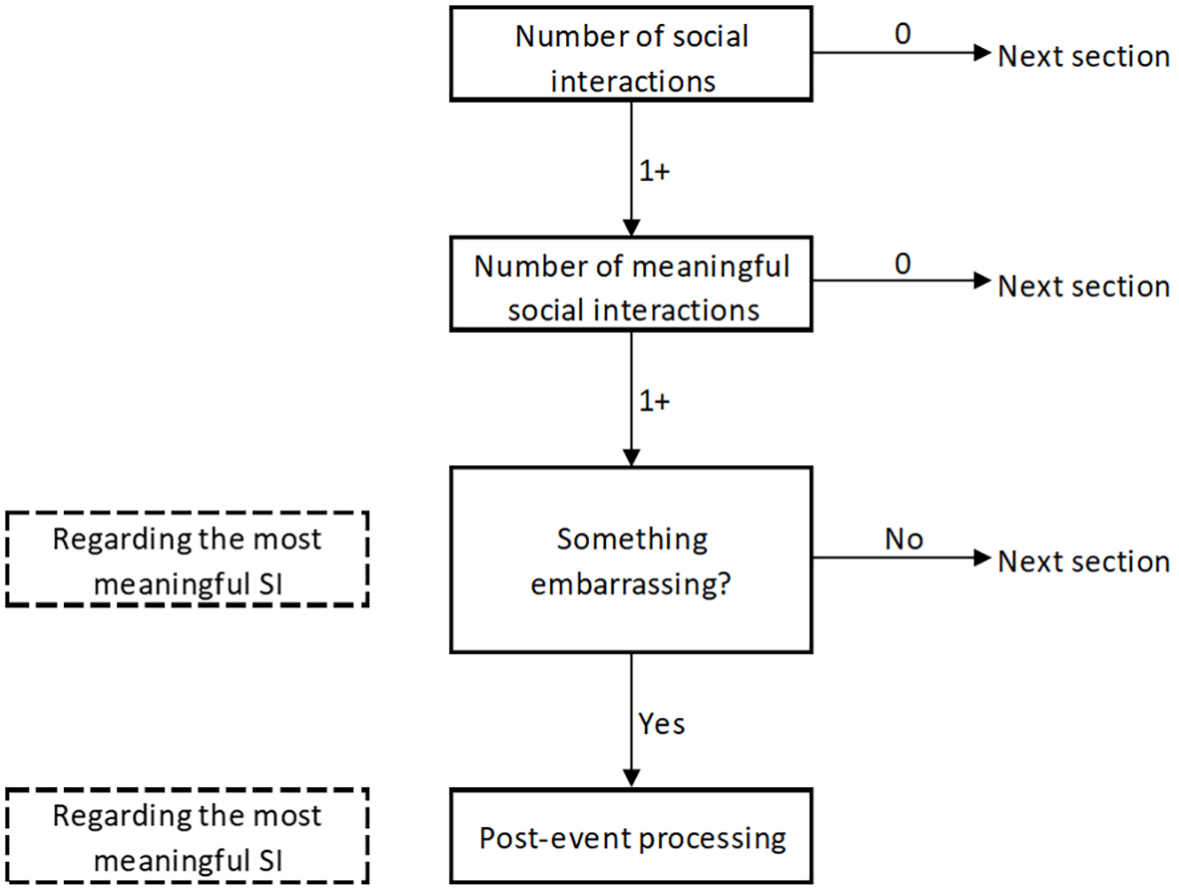
Survey Structure

Participants were asked about the number of Sis (*“Since the last inquiry, how many social interactions did you have?”)* and the number of meaningful Sis (*“Since the last inquiry, how many of your social interactions were meaningful for you?”)* since the last inquiry. They could indicate their answers on a scale from 0 = *none* to 6 = *more than five* (1 = *one SI*, 2 = *two SIs*, etc.). If they indicated having at least one meaningful SI, they were asked to report about one SI that was the most meaningful for them (from then on questions began with *“Regarding the most meaningful SI…”*). They were then asked whether they behaved in an embarrassing manner during that SI (“*Regarding the most meaningful SI, did you, in your own opinion, in some way behave in an embarrassing manner?*”). Only if they indicated doing something embarrassing, were they asked about the degree of PEP (for survey structure see Figure 1).

#### Social Interaction Anxiety Scale (SIAS)

The SIAS is an inventory developed to assess anxiety in SIs (Mattick & Clarke, 1998). It consists of 20 items that depict multiple socially anxious behaviours. The items are rated on a five-point scale. The German version of the SIAS (Stangier et al., 1999) showed high internal consistency (α = .89-.94) across SP and MDD, as well as high test-retest reliability (*r* = .92) across various samples.

#### Beck Depression Inventory II (BDI-II)

The BDI-II (Beck et al., 1996) is the most widely used measure of depression. It consists of 21 items depicting various dimensions of depression. The German version (Hautzinger et al., 2006) that was used in the present study showed sound psychometric properties, exhibiting a high internal consistency (Cronbach’s α = .92-.93) and a high test-retest reliability (*r* = .93).

### Procedure

In the overall study project, data were collected over two weeks with observations in seven-day intervals (Gloster et al., 2017). Time 1 occurred on the first day, Time 2 on the eight day and Time 3 on the 15^th^ day of the study. The ESM took place between Time 2 and Time 3. Both the SIAS and BDI-II were assessed as traits at time point 2 before giving out the smartphones (for the complete study design see Gloster et al., 2017).

Participants received a smartphone and were instructed in its use. They were shown how to operate the smartphone, how to recognize the signal tone and how to initiate a survey after a signal.

The ESM took place for seven days. Every day participants completed five surveys on the smartphone screen at fixed times, every three hours, meaning that participants could have completed a maximum of 35 (i.e. 7 x 5) surveys (Gloster et al., 2017). Prior to receiving the smartphone, participants could decide whether the first survey of the day would be at 10 a.m. or at 11 a.m. The survey would then start on all of the following days at that chosen time.

### Statistical Analysis

Data were analysed with Stata Statistical Software Version 14.2. (StataCorp, 2015). For the analysis of between-group differences in SIs, in indications of embarrassment and in the relative frequency of PEP (H1, frequency; exploratory analysis), random effects logistic regression analyses were conducted (Rabe-Hesketh & Skrondal, 2012). For these purposes, both PEP variables were recoded. If participants indicated having PEP in both items, the answer was coded with 1, and in the opposite case with 0. Also, the items assessing the number of overall and meaningful SIs were dichotomized (0 = 0, ≥ 1 = 1). To analyse the contributions of trait-social anxiety (SA) and trait-depression to PEP (H2), the SIAS and BDI-II scores were mean-centred and added as level-2 variables in the previous regression analysis. Additionally, we estimated via multilevel mixed effects linear regression analysis (H1, duration) whether groups differed regarding time spent thinking about the event (PEP, Item 1) and regarding time spent having difficulties to forget the event (PEP, Item 2). In all estimations, the variable indicating group-affiliation was dummy coded (controls = 0, MDD = 1, SP = 2) and used in the regression analysis as predictor. For comparisons of two groups, the group coded with the lower number was used as the reference group (controls in the case of controls vs. MDD and controls vs. SP; MDD in the case of MDD vs. SP). The mentioned analyses were conducted also for comparisons between the “pure” SP (without MDD, coded as 0) and MDD group (without SP, coded as 1) and the comorbid group (mixed SP/MDD; H3). The intercept was specified as random. Except for the linear regression analysis, odds ratios with corresponding 95% confidence intervals were calculated as the resulting measures. In all analyses, the *p*-value was set to .05.

## Results

Overall, the participants completed 91.8% of the EMA-assessments. There were no between-group differences in the response rate (see Supplemental Materials).

### SIs and Embarrassment

The controls differed from MDD and SP regarding the number of overall SIs, while there was no difference between MDD and SP. There were no between-group differences in the number of meaningful SIs (see Table 2). For a more detailed overview of results see Villanueva et al. (2020). The relative frequencies of embarrassing situations within the reported meaningful interactions were significantly higher in MDD and SP in comparison to controls, while there were no differences between MDD and SP. Also, we explored between-group differences in instances of repeated embarrassment on the same day. These were higher in MDD and in SP compared to controls, while MDD and SP did not differ (see Table 2).

### PEP After Embarrassing SIs (H1)

#### Frequency

When considering only the interactions in which participants felt embarrassed, participants indicated thinking repetitively about the interaction (PEP Item 1) in 95.68% of embarrassing SIs (controls: 96.67%; MDD: 96.07%; SP: 94.62%). Difficulties to forget the event (PEP Item 2) were reported in 94.02% of embarrassing SIs (controls: 86.67%; MDD: 93.82%; SP: 96.77%). There were neither differences between the groups in the relative frequency of repetitive thoughts (PEP, Item 1: controls vs. MDD, *OR* = 0.85, *p* = .888, 95% CI [0.09, 7.70]; controls vs. SP, *OR* = 0.58, *p* = .646, 95% CI [0.06, 5.69]; MDD vs. SP, *OR* = 0.69, *p* = .567, 95% CI [0.19, 2.47]), nor in the relative frequency of difficulties to forget the event (PEP, Item 2: controls vs. MDD, *OR* = 4.57, *p* = .170, 95% CI [0.52, 40.16], controls vs. SP, *OR* = 7.45, *p* = .123, 95% CI [0.58, 96.11], MDD vs. SP, *OR* = 1.62, *p* = .651, 95% CI [0.20, 13.50]). Due to elevated indications of embarrassment in SP and MDD compared to controls, it follows that PEP would also be higher in the clinical groups.

To account for imprecisions during the answer selection on the visual analogue scale (e.g. mistakenly marking a low number instead of a 0), we repeated the analyses while recoding the PEP variables as 0 when PEP ≤ 5% and when PEP ≤ 10%. However, no between-group differences were found. Results are available upon request.

#### Duration

The reported duration of PEP is presented in Table 3. There were no between-group differences.

### Controlling for Social Anxiety and Depression (H2)

#### Embarrassment in SIs

When trait SA was controlled, no differences between MDD and controls were found in indications of embarrassment. When trait depression was controlled, SIs were interpreted as embarrassing significantly more in SP compared to controls. The results are shown in Table 2.

#### PEP After Embarrassing SIs

The between-group differences in the frequency and duration of PEP remained non-significant even after controlling for levels of SA and depression of the individual. The results are presented in the Supplemental Materials.

#### Day-Level Embarrassment and PEP

We calculated day level embarrassment and PEP in the groups and we explored between-group differences. Controls differed significantly from MDD and from SP in each embarrassment and PEP (both variables), while there were no differences between MDD and SP (see Table 4).

**Table 2:** Between-Group Differences in Relative Frequencies of Indications of Overall SIs, of Meaningful SIs and of Embarrassment Within Reported Inquiries (N = 284)

Variable	Controls	MDD	SP	Controls vs. MDD			Controls vs. SP			MDD vs. SP		
	rf (%)	rf (%)	rf (%)	*OR*	p*	95% CI	*OR*	*p*	95% CI	*OR*	*p*	95% CI
Any SI^a^	80.4	74.0	72.6	**0.63**	**.006**	**[0.45, 0.87]**	**0.57**	**.010**	**[0.37, 0.87]**	0.90	.625	[0.58, 1.38]
Any meaningful SI^b^	84.9	85.3	85.2	1.01	.682	[0.73, 1.38]	1.09	.682	[0.72, 1.67]	1.09	.702	[0.71, 1.66]
Indications of embarrassment^c^	2.14	8.96	11.73	**4.78**	**< .001**	**[2.55, 8.96]**	**6.93**	**< .001**	**[3.35, 14.35]**	1.45	.213	[0.81, 2.60]
Repeated embarrassment^d^	3.33	14.04	12.90	**2.80**	**.005**	**[1.04, 5.91]**	**2.76**	**.006**	**[1.05, 6.20]**	0.21	.836	[-1.25, 1.54]
Differences in indications of embarrassment while controlling for	Trait social anxiety			1.86	.085	[0.92, 3.76]	–			–		
	Trait depression			–			**3.76**	**.001**	**[1.77, 7.98]**	**–**		

*Note*. Controls = Control subjects; MDD = major depressive disorder; SP = Social phobia; rf (%) = relative percentages.

^a^Frequencies are relative to the total sum of social interactions: 9105 (Denominators: Controls = 3868, MDD = 3747, SP = 1490).

^b^Frequencies are relative to the total sum of meaningful social interactions: 6965 (Denominators: Controls = 3111, MDD = 2772, SP = 1082).

^c^Frequencies are relative to the total sum of reports about the most meaningful social interaction: 4183 (Denominators: Controls = 793, MDD = 1986, SP = 1404).

^d^Frequencies are relative to the total sum of indications of embarrassment: 301 (Denominators: Controls = 30, MDD = 178, SP = 93).

*Significant differences are bold.

**Table 3:** Between-Group Differences in the Duration of Time Spent Engaging in Post-Event Processing (N = 284)

Variable	Controls	MDD	SP	Controls vs. MDD			Controls vs. SP			MDD vs. SP		
	*M* (*SE*)	*M* (*SE*)	*M* (*SE*)	*z*	*p*	95% CI	*z*	*p*	95% CI	*z*	*p*	95% CI
PEP, Item 1	35.50 (5.47)	43.57 (2.60)	40.05 (3.75)	1.33	.182	[-3.79, 19.94]	0.69	.493	[-8.45, 17.56]	-0.77	.440	[-12.48, 5.43]
PEP, Item 2	32.10 (5.72)	43.68 (2.78)	40.72 (4.05)	1.82	.069	[-0.88, 24.06]	1.23	.219	[-5.12, 22.37]	-0.60	.546	[-12.59, 6.67]

*Note*. PEP, Item 1 = Time spent thinking repetitively about the embarrassing event; PEP, Item 2 = Time spent having difficulties to forget the embarrassing event; Controls = Control group; MDD = major depressive disorder; SP = Social phobia.

**Table 4:** Between-Group Differences in Day Levels of Embarrassment and Post-Event Processing (N = 284)

Variable	Controls	MDD	SP	Controls vs. MDD			Controls vs. SP			MDD vs. SP		
	*M* (*SD*)	*M* (*SD*)	*M* (*SD*)	*z*	*p*	95% CI	*z*	*p*	95% CI	*z*	*p*	95% CI
Embarrassment	0.03 (0.20)	0.21 (0.55)	0.29 (0.60)	**5.16**	**< .001**	**[0.11, 0.25]**	**5.30**	**< .001**	**[0.16, 0.34]**	1.41	.16	[-0.03, 0.15]
PEP1	0.03 (0.19)	0.20 (0.54)	0.27 (0.59)	**5.09**	**< .001**	**[0.11, 0.24]**	**5.06**	**< .001**	**[0.14, 0.32]**	1.21	.22	[-0.03, 0.14]
PEP2	0.03 (0.18)	0.20 (0.54)	0.28 (0.58)	**5.10**	**< .001**	**[0.11, 0.24]**	**5.29**	**< .001**	**[0.15, 0.33]**	1.44	.15	[-0.02, 0.15]

*Note*. PEP, Item 1 = Time spent thinking repetitively about the embarrassing event; PEP, Item 2 = Time spent having difficulties to forget the embarrassing event; Controls = Control group; MDD = major depressive disorder; SP = Social phobia.

We also explored associations between embarrassment and both PEP variables on the day level. Both variables significantly predicted embarrassment: Repetitive thoughts, β = 0.58, *p* < .001, 95% CI [0.56, 0.60]; Difficulties to forget the event, β = 0.43, *p* < .001, 95% CI [0.42, 0.45].

### Controlling for Co-Morbidities Between MDD and SP (H3)

To investigate the contribution of co-morbidity, we divided the groups into patients with MDD and no SP as a co-morbid diagnosis (= *MDD/noSP*), patients with SP and no MDD as a co-morbid diagnosis (= *SP/noMDD*) and patients with mixed MDD and SP (= *mixed/MDD/SP)*. We then analysed differences between these groups in indications of embarrassment as well as in the duration and frequency of both PEP items. No between-group differences were found regarding any of these variables. Results are presented in the Supplemental Materials.

## Discussion

The findings highlight the high incidence of PEP in individuals with SP and MDD, as well as controls, whenever a situation is perceived as embarrassing. The comprehensive nature of PEP and its close ties to embarrassment are best reflected in its consistently high rates across all groups. At least 86% of all participants, irrespective of their diagnostic status, reported PEP following an embarrassing SI. The groups differed regarding neither its relative frequency nor its duration. These findings must be interpreted with caution, as we do not know the specific content of those repetitive thoughts in clinical groups and controls. While the clinical groups may have reinforced their dysfunctional cognitions, the controls might have focused more on coping with the embarrassing moment. However, while repetitive thinking about a recent embarrassing event seems to be common to all individuals, the more frequent indications of the event as being embarrassing in the first place might be specific for SP and MDD. Thus, we can argue that the repetitive thoughts or difficulties to forget the embarrassing moment are not unusual, but rather the contextual processes preceding and laying foundation for their emergence, like the higher occurrence of subjective embarrassment. This was evident in the repeated embarrassment and the day-levels of embarrassment as well.

One explanation may be that individuals with SP and MDD engage in misappraisals of the situation. Such misappraisals are driven by high SA, characteristic not just for socially anxious but depressed individuals as well (e.g. T. A. Brown et al., 2001), as between-group differences in indications of embarrassment dissipated after holding SA constant. This is in line with existing research of self-perception and cognitive biases related to SA. Individuals with elevated levels of SA scrutinise their behaviour and underestimate how well they appear to others (Mansell & Clark, 1999). They are especially sensitive to threat cues and are more likely to interpret ambiguous reactions as evidence of negative social evaluation (Heinrichs & Hofmann, 2001; Stopa & Clark, 2000).

An alternate explanation is that individuals with SP or MDD actually behave in more embarrassing ways due to a potential lack of social competence or due to the use of open or covert safety behaviours and concerns about their appearance (e.g. Moscovitch et al., 2013). Empirical data make this explanation, however, less probable as individuals with high social anxiety tend to be more biased in their evaluation of their own performance than in their social competence per se (Alden & Wallace, 1995; Stopa & Clark, 2000).

Accordingly, we can assume that heightened SA contributes to an event more likely to be perceived as embarrassing by the individual. However, once feelings of embarrassment are activated, they can produce subsequent ruminative patterns irrespective of the diagnostic status. When trait SA is low, the indications of embarrassment, and consequently PEP, are reduced to non-clinical levels. Nonetheless, because of the higher occurrence of repeated embarrassment and day-level embarrassment in the clinical groups, day-level PEP was also significantly increased compared to controls. We can draw on these findings to propose a model of PEP in MDD and to complement previous research on the formation of PEP in SP.

Considering our analyses, in MDD both depressive *and* socially anxious states function as catalysts for PEP, but only symptoms of SA are a prerequisite to experience PEP. Hence, the following cycle can be proposed: heightened levels of SA in MDD might lead to more social events being interpreted as embarrassing. Once embarrassment is experienced, the ongoing ruminations in depressed individuals, which are more general and encompass various areas of life (McEvoy et al., 2010; Nolen-Hoeksema et al., 2008), might include social encounters as a subject matter too, so that PEP arises. On the other hand, if SA is low in MDD, it can be argued that social events might drop out as a possible content of ruminations, thus reducing the frequency of PEP. However, it is not clear from our data what the content of these ruminations was, because only the frequency and duration of PEP was assessed. While the *quantity* of PEP might have been the same, just as with SIs in previous research (Baddeley et al., 2013; Nezlek et al., 2000), the “quality” (i.e. content, affectivity) might have differed. According to previous research, it is reasonable to assume that in MDD the content consists of interpersonal rejection and accompanying beliefs of being less valuable (Dill & Anderson, 1999; Gotlib et al., 2004; Segrin, 2000). To explore this possibility, additional research investigating the cognitive content of PEP in MDD is needed.

In relation to SP, our results imply that SA and the heightened probability of PEP are mediated through feelings of embarrassment. This is consistent with previous findings that negative self-perception mediates the relationship between SA and PEP (Perini et al., 2006). The present study expands those findings to other diagnoses, as well as to healthy individuals. On this basis, we can argue that SA is a marker that facilitates negative self-perception, which then enhances feelings of embarrassment and ultimately PEP.

A treatment approach for PEP could comprise interventions correcting for maladaptive interpretations that act as its precursor. Thus, by minimising the (mis-) perceptions of embarrassment during SIs, it can be argued that the probability of subsequent PEP might significantly be reduced. Another strategy would be meta-cognitive therapeutic interventions correcting for the subsequent ruminations (Wells, 2009).

Also, we found that patients with MDD or SP indicated less frequently having had *any* SI since the last inquiry than controls. This might reflect the social difficulties of the clinical groups (e.g. L. H. Brown et al., 2011; Chen et al., 2013). However, the groups did not differ in indications of *meaningful* SIs. This could reflect the importance of social values compared to other values for patients with MDD and SP. Patients tend to exhibit value-consistent behavior in social life areas, which could lead them into SIs that are meaningful to them (Wersebe et al., 2017).

### Limitations and Outlook

The question remains whether the contents of those ruminations were maladaptive as well, since we only inquired if repetitive thinking occurred and if individuals had difficulties forgetting the events. It is possible that the controls focused on coping with the event and reframing the embarrassing moment in a positive way, while the clinical groups focused on negative evaluation or self-worthlessness. To discriminate between controls and clinical groups, as well as between specific cognitive biases in SP and MDD, future research should include additional items exploring the content of ruminative thoughts.

An additional limitation is the use of only two items to measure PEP, which makes our assessment highly specific. Future studies should include a questionnaire that encompasses multiple dimensions of PEP and ideally a cut-off score for clinically significant severity of PEP. That would allow us to explore whether the incidence rates of PEP remain equally high in controls as in the clinical groups. It could as well be possible that the current PEP measure is not sensible enough to detect differences between clinical groups and controls. Even though we assessed the duration of PEP as well and did not find differences between groups, an option in future research could be the inclusion of multiple PEP measures.

Also, the nested structure of the survey allowed for explorations of PEP only within *the most meaningful* SI in which *also* feelings of embarrassment were experienced. This is due to the study being embedded within a large research project that explores a variety of transdiagnostic phenomena with multiple measures and across multiple disorders (Gloster et al., 2017). While this strategy provides an abundance of insights across multiple domains, some questions regarding PEP remain open. Most notably, it remains unclear how often PEP occurs across other SIs (vs. the most meaningful) during the day. Future ESM studies constructed specifically for the investigation of PEP should explore these research questions.

Lastly, as the present study put the importance of embarrassment forward, it would be intriguing to explore further emotion and thought patterns following embarrassing SIs. Since this goes beyond the scope of the present article, it should be also a matter of future ESM studies.

### Conclusions

The main conclusions of the study were that patients with SP and MDD had equal durations and frequencies of PEP as controls, but more frequent indications of embarrassment in meaningful SIs than controls. The indications of embarrassment were primarily driven by trait social anxiety.

The limitations notwithstanding, the investigation clearly demonstrated that SA and embarrassment (as a potential mediator) can be considered important psychological mechanisms behind PEP in SP and in MDD. By implementing ESM, responses are ecologically valid and less biased than in questionnaire or laboratory research.

## Supplementary Materials

The Supplementary Materials contain the following sections (for access see Index of Supplementary Materials below):

- Section X1 = Between-group differences in the occurrence of post-event processing after embarrassing social interactions after controlling for social anxiety and depression
- Section X2 = Differences in embarrassment, and the frequency and duration of post-event processing between the exclusive SP and MDD groups and the comorbid SP/MDD group
- Section X3 = Differences in completed EMA-assessments

10.23668/psycharchives.4429 Supplement 1 Supplementary materials to "Post-event processing after embarrassing situations: Comparing experience sampling data of depressed and socially anxious individuals"

ČolićJ.
LatyshevaA.
BassettT. R.
ImbodenC.
BaderK.
HatzingerM.
HoyerJ.

 (2020). Supplementary materials to "Post-event processing after embarrassing situations: Comparing experience sampling data of depressed and socially anxious individuals"
[Additional information]. PsychOpen. 10.23668/psycharchives.4429PMC964546936398063

## Author Note

Prof. Dr. A. Gloster and Prof. Dr. J. Hoyer planned and conducted this study in close collaboration and function as shared senior authors.
