# Supplementary material for: Post-Event Processing After Embarrassing Situations: Comparing Experience Sampling Data of Depressed and Socially Anxious Individuals
Source: Clin Psychol Eur. 2020 Dec 23;2(4):e2867. doi: 10.32872/cpe.v2i4.2867 (PMC9645469; doi:10.32872/cpe.v2i4.2867)
Supplement: Supplement 1 [file cpe-02-2867-s1.pdf]

Post-event processing after embarrassing situations: Comparing experience sampling data of depressed and socially anxious individuals

Supplemental material

Table X1. *Between-group differences in the occurrence of post-event processing after embarrassing social interactions after controlling for social anxiety and depression*

Controlling for		Reference Group	Group	*OR*	*p*	95% CI	
						LL	*UL*
SA	PEP 1	Controls	vs. MDD	0.39	.419	.043	3.70
	PEP 2	Controls	vs. MDD	1.30	.805	.162	10.44
Depression	PEP 1	Controls	vs. SP	0.57	.642	.055	6.01
	PEP 2	Controls	vs. SP	9.07	.116	.580	141.95

Note: PEP 1 = Post-event processing, item 1 ("repetitive thoughts about the event"); PEP 2 = Post-event processing, item 2 ("difficulties to forget the event"); Controls = Control subjects; MDD = Major depressive disorder; SP = Social phobia; SA = Social anxiety.

Table X2. *Differences in embarrassment, and the frequency and duration of post-event processing between the exclusive SP and MDD groups and the comorbid SP/MDD group (N = 284)*

	Only SP vs. only MDD			Only SP vs. mixed SP/MDD			Only MDD vs. mixed SP/MDD		
	Coefficient	*p*	95% CI	Coefficient	*p*	95% CI	Coefficient	*p*	95% CI
Embarrassment	OR = 0.70	.32	[0.35, 1.40]	OR = 1.12	.77	[0.52, 2.40]	OR = 1.59	.13	[0.87, 2.92]
PEP1 (frequency)	OR = 1.37	.71	[0.27, 6.90]	OR = 1.54	.63	[0.27, 9.00]	OR = 1.13	.88	[0.23, 5.43]
PEP2 (frequency)	OR = 0.26	.44	[0.01, 8.00]	OR = 0.23	.43	[0.01, 9.02]	OR = 0.89	.92	[0.08, 9.51]
PEP1 (duration)	Δ = 8.37	.12	[-2.03, 18.77]	Δ = 11.51	.05	[0.28, 22.73]	Δ = 3.14	.49	[-5.83, 12.11]
PEP2 (duration)	Δ = 2.81	.64	[-9.05, 14.66]	Δ = 5.14	.43	[-7.72, 18.01]	Δ = 2.33	.65	[-7.87, 12.53]

Note. PEP 1 = Post-event processing, item 1 (“repetitive thoughts about the event”); PEP 2 = Post-event processing, item 2 (“difficulties to forget the event”); Only SP = Participants with social phobia and without major depressive disorder; only MDD = Participants with major depression and without social phobia; mixed SP/MDD = Participants with comorbid social phobia and major depression.

X3. Differences in completed EMA-assessments

The controls completed 6.81% of all EMA-assessments, which differed neither from the MDD group (completion rate: 8.92%, *OR* = 1.32, p = .18, 95% CI [0.88, 1.99]) nor from the SP group (completion rate: 10.02%, *OR* = 1.58, p = .09, 95% CI [0.93, 2.69]). The MDD and SP group also did not differ: *OR* = 1.20, p = .50, 95% CI [0.71, 2.02]).
